# Immediate type hypersensitivity and late phase reaction occurred consecutively in a patient receiving ethambutol and levofloxacin

**DOI:** 10.1186/s13223-018-0237-x

**Published:** 2018-04-03

**Authors:** Yukihiko Kato, Yu Sato, Miho Nakasu, Ryoji Tsuboi

**Affiliations:** 1 Department of Dermatology, Tokyo Metropolitan Tama Medical Center, 2-8-29 Musashidai, Fuchu, Tokyo 183-8524 Japan; 2 Department of Respiratory Medicine, Tokyo Metropolitan Tama Medical Center, 2-8-29 Musashidai, Fuchu, Tokyo 183-8524 Japan; 3 Department of Dermatology, Tokyo Medical University, 6-7-1 Nishishinjuku, Shinjukuku, Tokyo, 160-0023 Japan; 4 Present Address: Tokyo Medical University Hachioji Medical Center, 1163 Tatemachi, Hachioji, Tokyo 193-0998 Japan

**Keywords:** Immediate type hypersensitivity, Late phase reaction, Anti-tubercular therapy, Drug eruption, Ethambutol

## Abstract

**Background:**

We experienced a rare case of immediate type hypersensitivity and late phase reaction to anti-tubercular therapy consisting of ethambutol and levofloxacin, which occurred in close succession, giving the appearance of a single, continuous reaction to one drug.

**Case presentation:**

The patient was a man in his 70’s who began therapy consisting of isoniazide, rifampicin, and ethambutol for pulmonary tuberculosis. Since the patient had a drug eruption within several hours after the start of his treatment, his reaction to ethambutol was assessed first among the three suspected drugs using an oral challenge test. Levofloxacin, which was not among the suspected drugs, was administered with ethambutol in order to avoid drug resistance resulting from the administration of a single drug. The patient experienced pruritus within 1 h. We observed a well-defined, edematous erythema with induration, which persisted for several days after the patient received the two drugs. Next, skin tests were performed with ethambutol and levofloxacin. The skin reaction to ethambutol and levofloxacin consisted of two different types of allergic reaction, a immediate type reaction and phase reaction.

**Conclusion:**

This is the first report of a late phase reaction and immediate type hypersensitivity occurring in quick succession in the same patient. Subsequent skin tests were able to prove the presence of these two different types of allergic reactions.

## Background

Although adverse drug reactions to first-line drug therapy against tuberculosis are not rare, drug eruptions resulting from these medications are still a major obstacle to treatment. We experienced a rare case of late phase reaction and immediate type hypersensitivity to anti-tubercular therapy consisting of ethambutol and levofloxacin, which occurred in quick succession in the same patient so as to appear simultaneous. This mixed skin reaction was later able to be identified two distinct skin reactions.

## Case presentation

The patient was a man in his 70’s who began antitubercular therapy for pulmonary tuberculosis consisting of isoniazide, rifampicin, and ethambutol. The patient noticed a pruritic, erythematous patch on the trunk, 16 h after beginning treatment on day 1. By the next day itchy, edematous erythema with slight induration and warmth but without excoriation was observed on the face, trunk, and proximal extremities. Administering a topical steroid and oral anti-histamine after discontinuing the antitubercular medication improved the dermatitis and left only pigmentation.

**Fig. 1:**
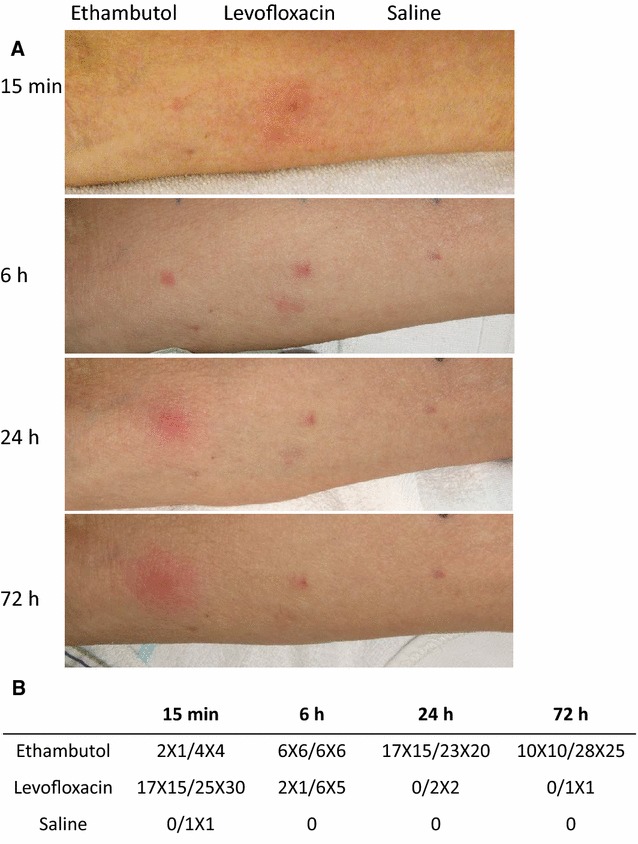
**A** Shows the results of the skin prick test using ethambutol, levofloxacin, and saline at 15 min, 6 h, 24 h, and 72 h after injection. **B** Shows the changes in the diameter of the wheal and induration/erythema over time

The patient did not remember whether he had ever taken levofloxacin or ethambutol, and his medical records showed no previous intake of those drugs. The patient’s reaction to ethambutol was assessed first among the three suspected drugs using an oral challenge test. Levofloxacin, which was not among the suspected drugs, was administered to the patient with ethambutol in order to avoid the risk of drug resistance resulting from the administration of a single drug [1, 2]. The patient presented urticarial lesions with pruritus within 1 h without other immediate type reaction such as, asthmatic reaction or rhinitic symptoms. We also observed a pruritic well-defined, edematous erythema with induration and warmth on the trunk and a small part of extremities which was not able to distinguish from urticarial lesions almost 8 h after he took the two drugs. These symptoms persisted for several days. Next, in order to identify the cause of the reaction, intradermal tests were performed with ethambutol and levofloxacin, both of which were crushed and diluted to 1% by weight with saline. A flare reaction occurred at the levofloxacin injection site 15 min after the test was administered and disappeared 6 h later (Fig. 1A, B). However, surprisingly, an indurative erythema with warmth at the ethambutol injection site emerged 6 h after the skin test, peaked at 24 h (Fig. 1A, B), and resolved on day 7. The ten healthy controls were negative for the skin test using ethambutol and levofloxacin 15 min to 72 h after test administration.

## Discussion

The skin reaction to ethambutol and levofloxacin consisted of two different types of allergic reaction, an immediate type reaction and late phase reaction (LPR) (Fig. 1A, B). Previous studies reported drug eruptions due to delayed type hypersensitivity to ethambutol and isoniazid [3] and immediate type hypersensitivity to rifampicin [4]. To the best of our knowledge, this is the first report of an immediate type hypersensitivity and late phase reaction occurring in quick succession in the same patient. Immediate hypersensitivity mediated by IgE is characterized by wheals and flaring at 15 min post-exposure while LPR exhibits erythematous, edematous, indurated lesions from 6 to 48 h post-exposure although swelling may continue to be observed 72 h later. The LPR itself is mediated by infiltrating inflammatory cells, such as mast cells, basophils, eosinophils, T cells, macrophages, and dendritic cells [5, 6] and is responsible for the chronic skin and bronchial inflammation seen in atopic dermatitis and asthma patients [7].

The mechanism causing consecutively, the nearly simultaneous occurrence of two different allergic reactions remains unclear. Bacsi et al. demonstrated an experimental model of allergic conjunctivitis in which pollen-mediated oxidative stress intensified immediate hypersensitivity and a late-phase reaction [8]. Kitagaki et al. reported that an immediate-type hypersensitivity reaction was followed by a late phase reaction after repeated epicutaneous application of an antigen in a mouse model [9]. However, such studies cannot account for the phenomenon observed in this case because the late phase reaction was observed independently from the immediate reaction and because the antigen or causative agent in immediate type hypersensitivity may not have been the same as that in the late phase response.

Some drugs like ciprofloxacin or atracurium are known to induce pseudo-allergic reactions by mast cell activation via Mrgprb2 [10]. Aspects of the immediate type reaction to the drugs in the present case might be explained by such a pseudo-allergic reaction. Cross sensitivity between ciprofloxacin and levofloxacin causing an immediate hypersensitivity reaction has been reported [1]. It is possible that the patient had previously been treated with ciprofloxacin. Furthermore the skin manifestation in this case might not be reflected with the skin test accurately.

Cross-reactivity might explain the unusual features of this case. However, cross reactivity usually manifests as one type of allergy. Furthermore, cross reactivity between ethambutol and levofloxacin has never been reported. These findings underscore the fact that drug eruptions are not necessarily caused by a single agent and that multiple types of allergic reaction may occur consecutively in a single case. In the present case the mixed drug eruption, which resembled a single drug eruption, was able to confirm the presence of two different types of allergic reaction, an LPR and immediate type hypersensitivity, using subsequent skin tests.

## Abbreviation

LPR: late phase reaction
